# Efficient machine learning of solute segregation energy based on physics-informed features

**DOI:** 10.1038/s41598-023-38533-8

**Published:** 2023-07-15

**Authors:** Zongyi Ma, Zhiliang Pan

**Affiliations:** Guangxi Education Department Key Laboratory of Microelectronic Packaging and Assembly Technology, School of Mechanical and Electrical Engineering, Guilin University of Electronic Technology, Guilin, 541004 Guangxi China

**Keywords:** Theory and computation, Metals and alloys

## Abstract

Machine learning models solute segregation energy based on appropriate features of segregation sites. Lumping many features together can give a decent accuracy but may suffer the curse of dimensionality. Here, we modeled the segregation energy with efficient machine learning using physics-informed features identified based on solid physical understanding. The features outperform the many features used in the literature work and the spectral neighbor analysis potential features by giving the best balance between accuracy and feature dimension, with the extent depending on machine learning algorithms and alloy systems. The excellence is attributed to the strong relevance to segregation energies and the mutual independence ensured by physics. In addition, the physics-informed features contain much less redundant information originating from the energy-only-concerned calculations in equilibrium states. This work showcases the merit of integrating physics in machine learning from the perspective of feature identification other than that of physics-informed machine learning algorithms.

## Introduction

An alloy is either a metallic compound or a solid solution composed of two or more elements, with one element as solvent or matrix and others as solute. The solute atoms may distribute either uniformly inside crystalline grains or preferentially into grain boundaries, a phenomenon known as solute segregation. As an efficient way to modulate the properties of nanostructured materials, solute segregation can inhibit grain growth by reducing the grain boundary (GB) energy^1,2^, increase the strength by stabilizing GBs^3,4^, improve the ductility by inducing the formation of toughening amorphous intergranular films^5,6^, or suppress the shear localization by forming solute-rich clusters^7^. The propensity of solute segregation is determined by the segregation energy^8^ that varies with numerous segregation sites and can be calculated through first-principles calculations^9–11^ or atomistic simulations^12^. While the former is limited by computational cost resulting from massive calculations of many possible segregation sites and the latter by accuracy, machine learning was brought out to do the balance based on data obtained from the two approaches^13^.

Like any other modeling technique, the general preference for machine learning is accuracy, an indicator describing how well a model identifies the relationship between features and labels. But this is not the only preference and many times computational cost is a concern. In the modeling of solute segregation energy, for example, there is a huge number of possible segregation sites. For each site, we need to extract features and predict the segregation energy using machine learning. While the computation cost for each site is negligible, that for all possible sites is not. Therefore, appropriate features need to be identified to balance the accuracy and the computational cost.

The segregation energy at a specific site is determined by the surrounding local atomic environment. Given many well-developed algorithms able to handle various problems, the main challenge with machine learning is to choose appropriate features^14^. While the relative coordinates of all neighboring atoms are not rotation and permutation-invariant and thus are not feasible features appropriate for the prediction of atom energy^15^, three types of features were usually extracted from the local atomic environment to make machine learning practical. The first is geometric features obtained by structural analysis. While performing machine learning of the segregation energy of six solute elements into various Al GBs, 19 geometric features can give a root mean square error (RMSE) varying from 0.04 to 0.13 eV, depending on the solute types^13^. The second is energy-based features such as atomic energy, interatomic force, stress, etc. Messina et al. combined these features and some geometric ones, 21 features in total, reaching an RMSE as low as 0.00621 eV when modeling Al segregation energy in Mg GBs^16^. The third type of feature is pure mathematical parameters^15,17^. These features have been widely used to develop machine learning interatomic potentials^18^. Typical examples include a smooth overlap of atomic positions (SOAP) potential^19^ and spectral neighbor analysis potential (SNAP)^17,20,21^. The SOAP feature is extracted by projecting the local atomic environment of an atom based on spherical harmonics and radial basis functions^15^. The SNAP is a machine learning potential that expresses the atomic energy as a linear function of bispectrum components, or in other words, SNAP features extracted by directly projecting the local atomic environment onto a basis of hyperspherical harmonics^17^. Wagih et al. extracted more than 1000 SOAP parameters, giving an RMSE of 0.04 eV while modeling the Mg segregation energy in an Al polycrystalline GB network^22^.

The few literature works put together any features that could affect the segregation energy, regardless of feature dimensions. As long as enough relevant features are included, the model can always give a decent prediction given an appropriate algorithm. However, features obtained this way might be dependent on each other or contain too much redundant information, causing a curse of dimensionality while performing machine learning, incurring unnecessary computational cost while extracting so many features from numerous segregation sites, and thus posing a great challenge to the application of these models. Here, we took a biased strategy by only extracting physics-informed (PI) features that have an undoubted effect on the segregation energy based on solid physical understanding. It is shown that merely three PI features can model the segregation energy with accuracy comparable to that using many more features in the literature work or that using many more SNAP features in this work. We attributed the excellent performance to that the PI features correlate to the segregation energy through different physical mechanisms and contain much less redundant information that is otherwise inherent to the SNAP features.

## Results and discussion

### Data preparation

**Figure 1:**
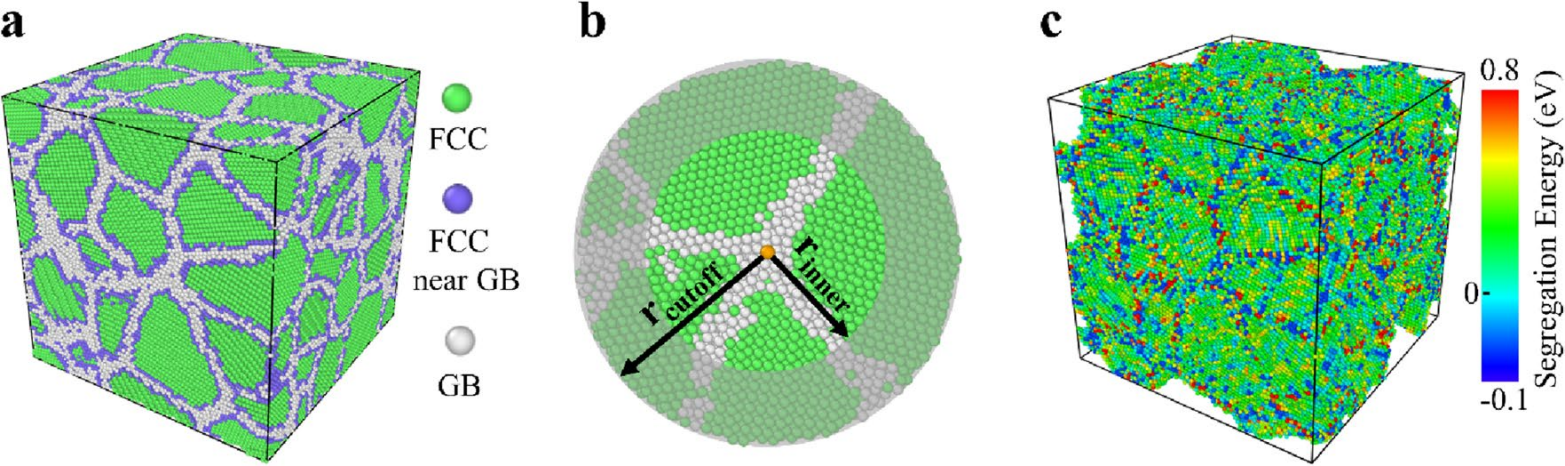
Atomic configurations used for preparing machine learning data. (**a**) A 20 × 20 × 20 nm^3^ polycrystalline Ag after annealing and energy minimization, containing 40 randomly oriented grains of ~ 7 nm in diameter, with face-centered cubic (FCC) atoms colored green, GB network atoms white, and neighboring FCC atoms blue. Atoms inside the GB network and the neighboring FCC atoms are considered possible segregation sites. (**b**) The local atomic environment around a segregation site based on which the segregation energy at this site is calculated. (**c**) The distribution of the calculated Ni segregation energy inside the Ag GB network and the neighboring crystalline atoms shows a segregation tendency at the majority of the selected sites.

Machine learning modeling consists of three general steps: data preparation, model training, and model testing (Fig. S1). Machine learning data should be representative of the sample space, a universal set of all possible segregation sites in this work. While the segregation could happen anywhere inside a GB network common in a realistic alloy, the literature usually used bicrystals when preparing the data^13,16,23^, where the possible sites are limited within the single GB inside the configuration. Even though many such configurations can provide a variety of GBs containing various segregation sites, the obtained data is yet a sufficient sampling for a common GB network due to the exclusion of triple junctions that are more preferable segregation sites^24^. Here we used a polycrystalline configuration containing 40 randomly oriented grains for each alloy system studied here, with an average grain size of ~ 7 nm (Fig. 1a). Each atom in the GB network, including GBs, triple junctions, and vertices, is considered as a possible segregation site. In addition, the neighboring crystalline atoms of the GB network, where segregation also happens, are considered possible sites as well^16,25^.

The local atomic environment is defined as the neighboring atoms within a sphere of radius ${r}_{cut}=3 \mathrm{nm}$ centered at a segregation site (Fig. 1b). The segregation energy is calculated as $\Delta {E}_{seg}^{sol}=\left({E}_{site}^{sol}-{E}_{site}\right)-\left({E}_{sgl}^{sol}-{E}_{sgl}\right)$, with a positive value indicating a segregation tendency. Here ${E}_{site}^{sol}$ and ${E}_{site}$ are the respective total energy of the atomic configuration shown in Fig. 1b after and before the centered matrix atom is replaced by a solute atom, whereas ${E}_{sgl}^{sol}$ and ${E}_{sgl}$ are the respective total energy of a reference single crystal after and before one host atom is replaced by a solute atom^12^. Details on how to calculate the segregation energy are presented in the Methods section. Figure 1c shows the distribution of the calculated Ni segregation energy within the GB network of a polycrystalline Ag. Most of the sites in the GB network, including the neighboring crystalline atoms, possess positive segregation energy and thus act as preferable segregation sites for Ni. But many sites inside the GB network also have negative segregation energy, showing an anti-segregation tendency.

### Identification of PI features

The PI features were identified based on physical understanding. The first PI feature is the atomic volume at a segregation site usually calculated with Voronoi analysis as the volume of a polyhedron enclosed by the faces perpendicular to and bisecting the lines between the segregation site and its neighboring atoms^26^. This feature indicates the interatomic distance between the segregation site and the surrounding nearest neighbors and has a strong effect on the solute binding energy^9^. Atomic volume suitable for the solute atom size tends to have high binding energy and is favorable for segregation, while that too small/large tends to decrease the binding energy and is not favorable for segregation. It is therefore highly expected and has been confirmed that the atomic volume is strongly relevant to the segregation energy^23^. Atomic volume only tells information about the nearest neighboring atoms. The information about the atoms further away can be described by a disorder factor. This PI feature measures how far the structure of a local atomic environment is away from a perfect crystalline structure or how close it is to a completely disordered structure, i.e. glassy state or amorphous structure. Atoms sitting in a perfect crystal environment will have a disorder factor of zero, while those in an amorphous structure will have a very large value. Both theoretical prediction and experimental observation showed that solute segregation and disordering transition promote each other at GBs^27–29^. Atomistic simulations^25^ also showed that amorphous GB structures have the highest solute composition, suggesting that the sites at highly disordered atomic environments tend to possess high segregation energy. The third PI feature is the atomic energy of the matrix atom at a site. Matrix atoms, or in other words, solvent atoms, with higher energy are less stable and more likely to be replaced by solute atoms and tend to possess higher segregation energy. For comparison, SNAP parameters were also extracted from the local atomic environment^17^. Designed to accurately model machine learning interatomic potentials, SNAP parameters are expected to give appreciable accuracy while modeling the solute segregation energy.

### Performance of PI features

**Figure 2:**
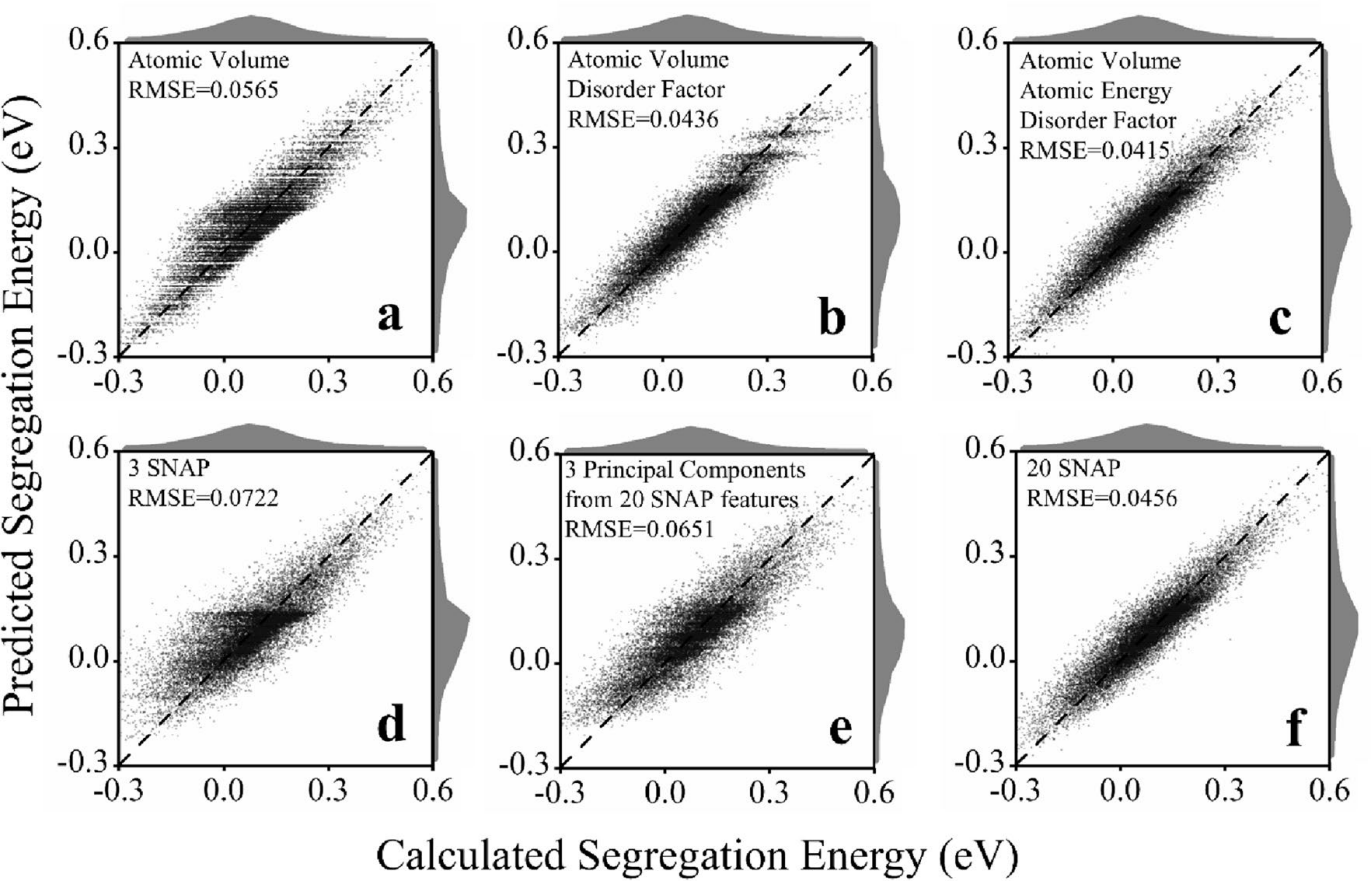
Validation of machine learning modeling on AgNi alloy using random forest algorithm. The statistical distribution of the calculated and predicted segregation energy is also shown. PI features used either separately or collectively (**a–c**) outperform SNAP features (**d–f**) with or without further dimensionality reduction, as indicated by the RMSE.

**Figure 3:**
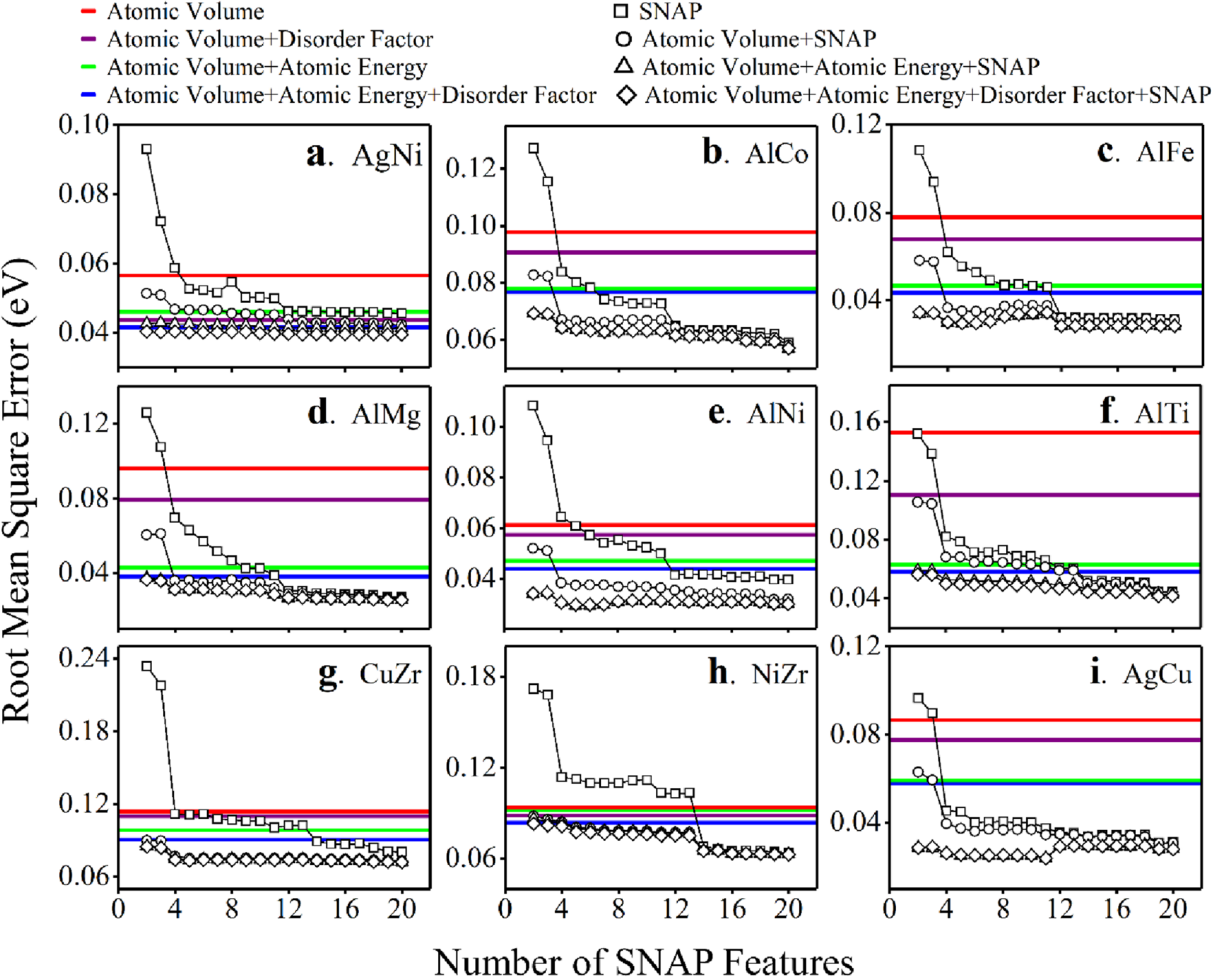
The performance of PI features on nine alloy systems using a random forest algorithm. PI features are much more accurate than the same number of SNAP features while used separately, significantly decrease the number of SNAP features for the same accuracy while used as an addition to SNAP features, and have no overfitting issues that the SNAP features suffer a lot.

Random forest algorithm was first used to do machine learning on a AgNi alloy system, with Fig. 2 showing that the three PI features, used either separately or collectively, outperform the SNAP features significantly. Atomic volume itself already gives an RMSE of 0.056 eV (Fig. 2a), more accurate than similar modeling of an AlMg system that also used atomic volume as the only feature but with training data obtained from bicrystals^23^. Adding a disorder factor into the feature set can reduce the RMSE to 0.0436 eV (Fig. 2b). Putting the three PI features together eventually reduces the RMSE to 0.0415 eV (Fig. 2c). In contrast, three SNAP features only give an RMSE of 0.0722 eV (Fig. 2d), even much higher than that based only on the atomic volume. The predicted statistical distribution of the segregation energy shifts towards a low-energy regime due to the poor performance. While increasing the number of SNAP features decreases the RMSE (Fig. 3a) and 20 SNAP parameters give an RMSE of 0.0456 eV (Fig. 2f) comparable with the results in the literature work^13,22^, this RMSE is only as good as the RMSE based on atomic volume and atomic energy (Fig. 3a), still higher than that based on the atomic volume and disorder factor, not to mention the RMSE based on all PI features.

The PI features also outperform the SNAP features on eight other alloy systems in that the same number of PI features always gives lower RMSE than the same number of SNAP features (Fig. 3), with the extent depending on alloy systems. The atomic volume itself behaves as well as 2 SNAP features in AlTi alloy but 13 SNAP parameters in NiZr alloy; The RMSE of all PI features is only as low as three SNAP parameters in AgCu alloy but 18 SNAP parameters in AlNi alloy.

**Figure 4:**
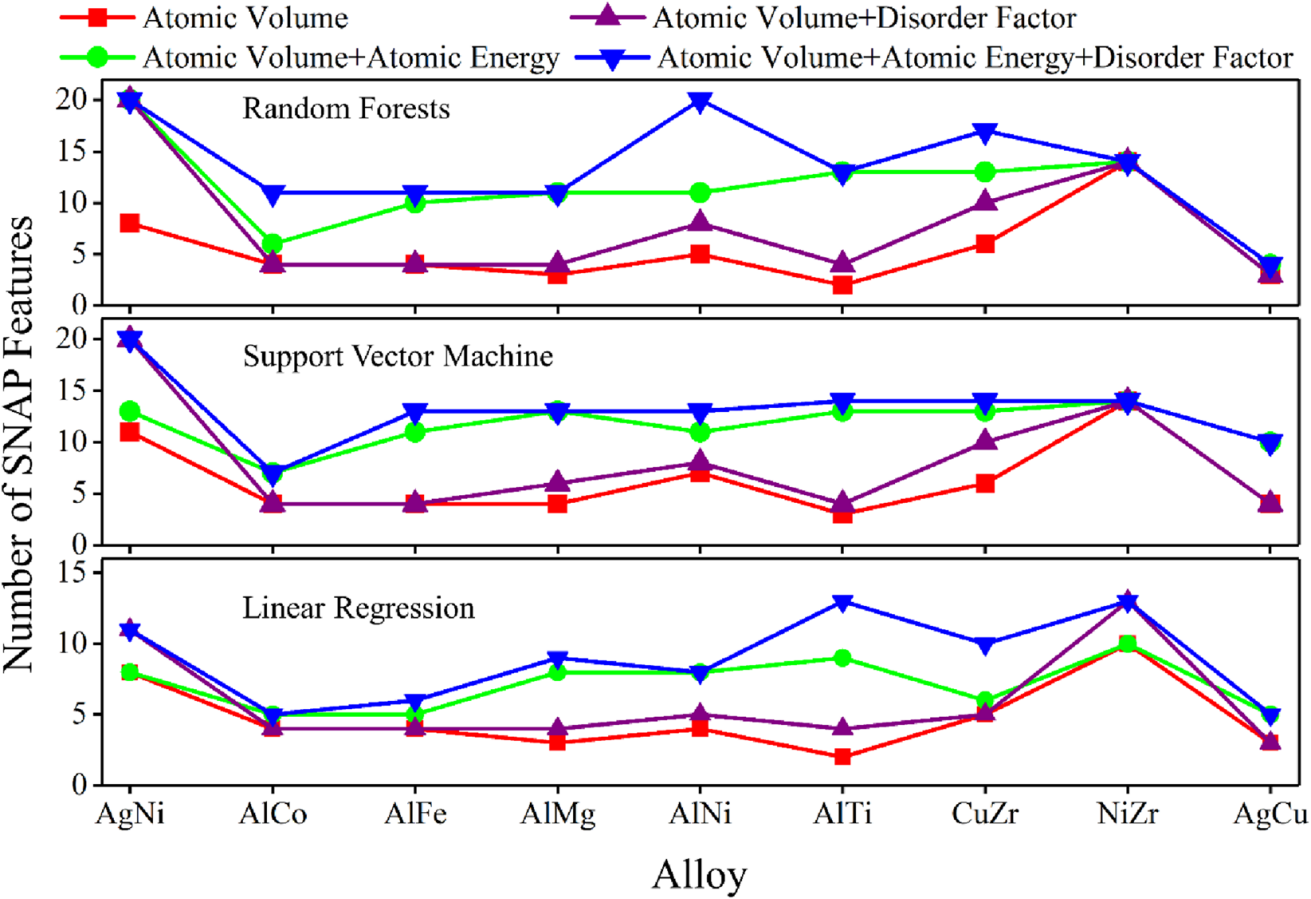
The performance of PI features using different machine learning algorithms on nine alloy systems. The number of SNAP features outperformed by PI features depends on the machine learning algorithms and alloy systems, with all PI features together always giving the best results.

The PI features continue excelling when using support vector machine and linear regression algorithms, albeit with different performance (Fig. 4). In the AgNi alloy system for example, atomic volume itself behaves better than four SNAP features using random forest and linear regression but 11 SNAP features using support vector machine. All three features work better than 20 SNAP features using random forest and support vector machine algorithms but only better than nine SNAP features while using linear regression algorithm. The 20 SNAP features behave the best with the linear regression algorithm because the atom energy of both the matrix and solute atoms at a local atomic environment is designed to be a linear function of these features^17^.

It is worth noting that SNAP features were added to the feature set in the as-output order from atomistic calculations while investigating the accuracy versus the number of SNAP features (Fig. 3, Figs. S2, S3). One may argue that the performance of PI features might result from a possible weak correlation of the preceding SNAP features to the segregation energy. To rule out this possibility, we sorted the SNAP features from the greatest to the least relevance to segregation energy, as indicated by a correlation matrix heat map (Fig. S4), and redo the investigation for AgNi alloy. Results (Fig. S5) similar to Fig. 3a, Figs. S2a, and S3a are obtained for the three respective algorithms, indicating that the performance of PI features does not strongly depend on the order of SNAP features.

**Figure 5:**
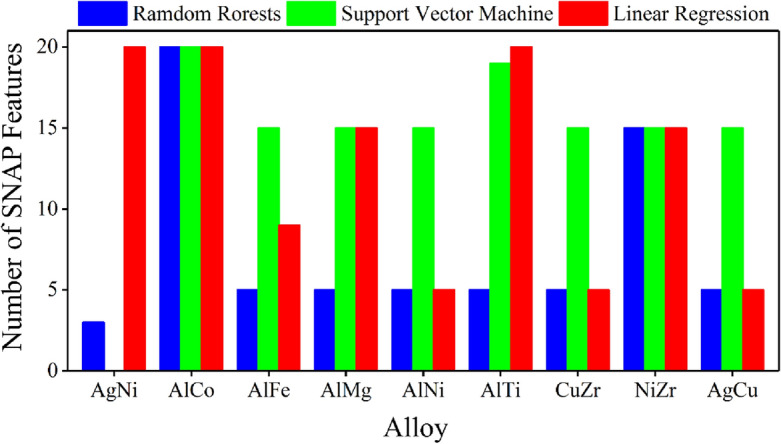
The number of SNAP features required to achieve the best machine learning accuracy when combined with PI features. The performance depends on machine learning algorithms and alloy systems.

Since both types of features contain information on the segregation energy, it would be interesting to put them together in machine learning. Figure 3 and Figs. S2 and S3 show that the addition of PI features, either separately or collectively, significantly improves the performance of SNAP features, in that the combined feature set requires much fewer SNAP features than pure SNAP parameters to reach the same accuracy. In AgNi alloy for example, using random forest algorithm, two SNAP features give an RMSE of ~ 0.095 eV (Fig. 3a), adding atomic volume can significantly reduce the RMSE to ~ 0.052 eV, comparable to that of 11 SNAP parameters. Further addition of atomic energy reduces the RMSE to a value lower than that from 20 SNAP features. It works even better by adding all three PI features to the two SNAP parameters. In addition, the lowest RMSE is always achieved by the addition of PI features, with only a few SNAP features in many cases (Fig. 5). For example, the lowest RMSE in AgNi alloy is achieved by adding PI features to 20 SNAP features with linear regression but only three SNAP features with random forest algorithm. PI features can even do the best without SNAP features while using a support vector machine algorithm.

Conventional wisdom in the literature to achieve accurate machine learning modeling is to include as many features as possible so that any possible information about the segregation energy is included in the training data^13,16,22^. This strategy may not live up to the expectations since too many features might lead to overfitting, a manifestation of the curse of dimensionality. In this regard, the efficiency of PI features also manifests itself by hardly showing overfitting that SNAP features suffer a lot. Figure 3, Figs. S2, and S3 show that the RMSE usually decreases with the number of SNAP features, whether used separately or combined with the PI features. This decrease, however, is not monotonic sometimes, indicating an overfitting problem with too many features (Fig. 3c, i, Figs. S2c, d, e). Overfitting is unclear in the literature due to the lack of comparative study but should be highly likely due to a large number of lumped features. In contrast, PI features almost have no overfitting, with the only exception in AlMg alloy while using support vector machine algorithm, that atomic volume plus atomic energy works slightly better than all PI features.

### Source of redundant information

The performance of PI features over SNAP features strongly suggests that the information of segregation energy is densely compacted in the former as we expected, but spread more widely in SNAP features and probably in the many lumped features in the literature work. In other words, SNAP features contain more redundant information than PI features. On one hand, SNAP parameters are developed to model interatomic potentials in classical atomistic simulations and are supposed to keep the information of the atomic energy, the interatomic force, and the stress tensor of the central atom while extracted from a local atomic environment^17^, where the argument should also hold for SOAP parameters^15^. In the calculation of solute segregation energy, however, the only useful information is the energy term. Information about the interatomic force and the stress tensor is inherently retained in SNAP parameters as redundant information in the machine learning data. On the other hand, the segregation energy is calculated based only on equilibrium local atomic configurations. The interatomic forces are all zero or close to zero within the reference crystalline configuration and the atomic configurations of possible segregation sites. The force information stored in SNAP features is not only useless but also constant zero. Therefore, the local atomic configuration space used in the segregation energy calculations is only a special subset of the universal atomic configuration space that is supposed to be described quite accurately with SNAP or SOAP parameters.

**Figure 6:**
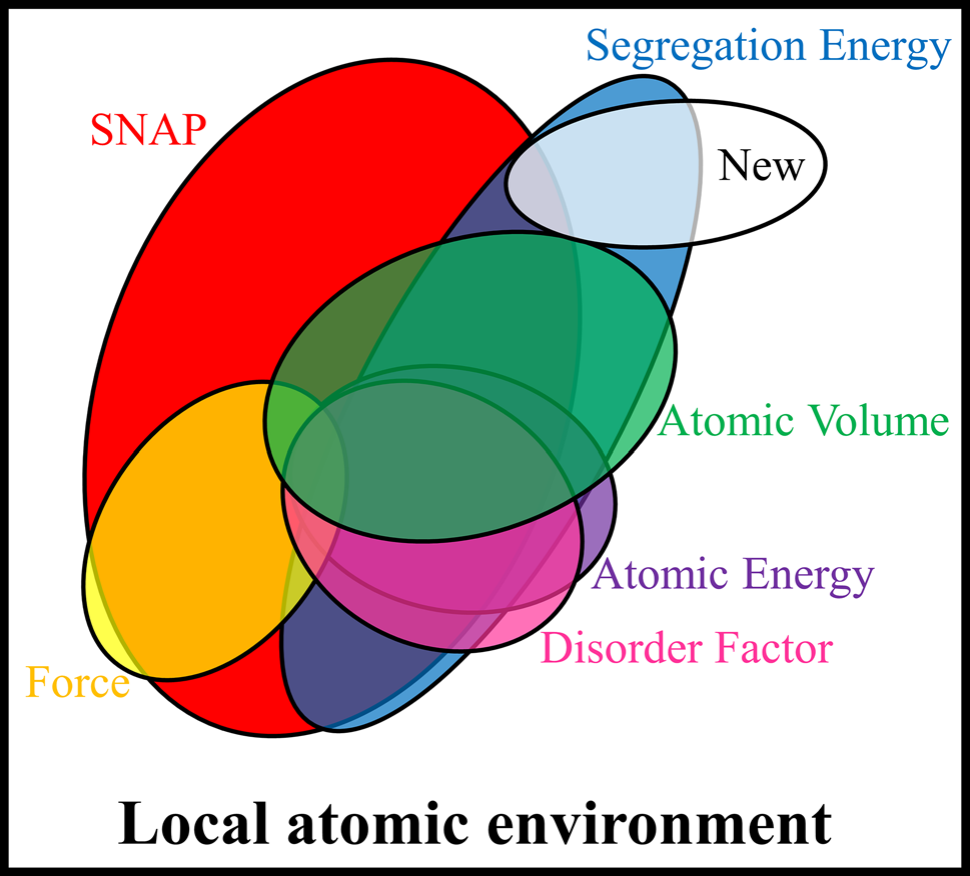
The Venn diagram demonstrates the interrelationship between different features and solute segregation energy. As the universal set, the local atomic environment contains all the information of the site of interest, meaning all features listed in this diagram are extracted either directly or indirectly from the local atomic environment. SNAP features overlap with the segregation energy, explaining why machine learning based on SNAP features gives decent accuracy while modeling the segregation energy. The large area of the SNAP set that does not overlap with the segregation energy set represents the redundant information inherent to SNAP features. The sets of atomic volume, atomic energy, and disorder factor overlap with the segregation energy sufficiently, explaining the excellent machine-learning performance of the three features. Whenever possible, more new features can always be extracted from the local atomic environment to cover more information on the segregation energy, as demonstrated by the new feature set overlapping with the segregation energy set.

The correlation between the local atomic environment, PI features, SNAP features, segregation energy, etc. can be visualized in a Venn diagram (Fig. 6). All of the closed curves are within the rectangle, meaning that all information is contained in the local atomic environment. Most of the force set is overlapped with the SNAP set, implying that SNAP features can be used to predict the interatomic forces. The segregation energy set is also largely overlapped with the SNAP set, explaining why SNAP features work well in the modeling of segregation energy. However, the overlap only accounts for a smaller area of the SNAP set. The other larger area does not contain information on the segregation energy and acts as redundant information. Therefore, using SNAP features to model segregation energy is like putting a fine timber to petty use, working well but wasting many feature functions and computational resources.

### The merit of physics-informed feature identification

One natural strategy to reduce the redundant information is to perform dimensionality reduction, for example, using principal component analysis, on the SNAP features. The accuracy of three principal components (Fig. 2e) is higher than that of three SNAP features (Fig. 2d) but lower than that of the 20 SNAP features (Fig. 2f), a fair compromise between accuracy and feature dimension. However, the three principle components still underperform PI features significantly. The accuracy is even lower than that of the atomic volume itself, not to mention all PI features. This inferior performance can be attributed to possible information loss during the feature extraction and subsequent dimensionality reduction process. First, extracting SNAP features from the local atomic environment may lose information, disordering state (Fig. S6) as an example, about segregation energy, resulting in errors in machine learning, even though the SNAP parameters are developed to model interatomic potentials. Dimensionality reduction can condense more information onto the principle components but cannot recover the information already lost when extracting SNAP features. Secondly, dimensionality reduction from SNAP features to principle components will also lose information, as confirmed by that the accuracy of the three principal components is still lower than that of the 20 SNAP features.

In contrast, PI features are directly extracted from the local atomic environment. Despite the inevitable information loss, the information about segregation energy is largely retained by always identifying the important features based on physical understanding. Secondly, each separate feature affects the segregation energy through different physical mechanisms, considerably ensuring feature independence sought by a successful feature extraction method. For example, both atomic energy and disorder factor are weakly correlated with atomic volume (Fig. S4). Though the atomic energy and disorder factor are more strongly correlated, indicating that a disordered structure tends to have higher atomic energy, the different physical mechanisms still ensure that the two features contain some different information about the segregation energy and the combination of the two features contains more information than each separately, as indicated by that all three PI features almost always give the best prediction of segregation energy. Thirdly, identified based on physical understanding, the PI features help pinpoint the important mechanism for the propensity of solute segregation, while the pure mathematical SNAP or SOAP features do not due to the lack of explicit physical meaning. Fourthly, in addition to the above demonstration that fewer PI features and thus less computational cost are required to perform machine learning than SNAP features, the extraction of a few PI features also requires much less computational resources than SNAP features since both feature extraction and further dimensionality reduction are computationally expensive. Lastly, extracted directly from the local atomic environment, new features (Fig. 6) can always be added to further improve the accuracy with additional computational cost only from the new features, while adding more SNAP features usually requires starting over the whole extraction process and at least doubles the already-high computational cost.

The above comparison showcases the merit of identifying features directly from the local atomic environment based on physical nature. Feature extraction or selection is probably the biggest challenge for machine learning. First, the features should contain as much information on the segregation energy as possible to ensure machine learning accuracy. Second, the features should contain as little redundant information as possible to reduce the computational cost. Last, the features should contain as little overlapped information as possible to further reduce the computational cost. Inappropriate features or too many ones may contain too much redundant or overlapped information, leading to poor model performance and/or high computational cost. Fortunately, modeling of segregation energy is a physical problem. One should always start by identifying the most important features that contain the most information about segregation energy by making the most of the physical nature. This strategy is like the one used in first-principles calculations based on density functional theory: first calculating the exchange–correlation energy based on local electronic density and then improving the accuracy by further considering the gradient^30,31^. By making sure the inclusion of important features and adding more relevant features if necessary, one can quickly approach sufficient accuracy with the least number of features. Otherwise, recklessly relying on the power of machine learning will solve the problem but likely with reduced efficiency.

## Outlook

The importance of integrating physics into machine learning has been demonstrated by the development of physics-informed machine learning algorithms (deep neural networks, kernel-based regression networks, etc.) while simulating multiphysics problems^32^. This work demonstrates the importance from a different perspective, i.e. feature identification, by showing that PI features have clear advantages over SNAP features. While the merit manifests itself on all of the nine alloy systems, PI features work best on the AgNi system, the interatomic potential of which happens to be the only potential developed by explicitly including solute segregation energy obtained through first-principles calculations in the potential fitting process^12^. It is thus highly likely that the solute segregation energy in this system is more accurate than that in eight other alloy systems. We, therefore, attribute the best performance to the data authenticity for this system and expect better performance if all data is prepared through first principles that by far give the most accurate calculations.

## Methods

The machine learning data of nine binary alloy systems was prepared through atomistic simulations using a Large-scale Atomic/Molecular Massively Parallel Simulator (LAMMPS)^33^, with embedded-atom-method or Finnis–Sinclair interatomic potentials^12,34–41^ describing the interatomic interactions. For each system, we first created a 20 × 20 × 20 nm^3^ polycrystalline atomic configuration of the matrix element containing 40 randomly oriented grains using the Voronoi tessellation method, with periodic boundary conditions applied in all three directions. The as-created model was first annealed for 50 ps at 300 K and zero pressure, with an integration time step of 1 fs. Then the total potential energy of the annealed configuration was minimized with a conjugate gradient method. The SNAP features and the potential energy of each atom were also calculated simultaneously at the last step of energy minimization.

A separate Fortran program was then used to calculate the disorder factor of each atom in the equilibrated configuration. The disorder factor of an atom was mathematically defined as the norm of the difference between the discrete radial distribution functions of the local atomic environment and a perfect single crystal. Based on this definition, the disorder factor of an atom in a single crystal will be zero. Any deviation will increase the disorder factor.

The equilibrated atomic configuration was finally visualized and analyzed using the open-source visualization tool OVITO^42^, where the atomic volume of each atom was calculated with Voronoi analysis. Common neighbor analysis^43^ was also used to identify the crystal structure of each atom, based on which ~ 0.2 million atoms within or close to GBs were selected as possible segregation sites (Fig. 1a). To calculate the segregation energy at these many sites with acceptable computational cost, we only considered atoms less than 3 nm away from each specific site, i.e. the local atomic environment and the atoms at the segregation site. While calculating the total energy before and after a solute atom decorating the site using a conjugate gradient method, the outer layer of atoms (shaded area in Fig. 1b) was always fixed to ensure the selected atoms behave as if in the whole sample and there is no significant structural change during the equilibration process after decoration.

The machine learning was performed using the open-source Python module scikit-learn^44^, where 90% of the whole data was used as training data and 10% as testing data. For the random forest algorithm, the number of decision trees is (5, 10, 20, 50, 100, 200), the depth of the maximum tree is (3, 5, 7), and the maximum number of features considered in the division of decision trees is (0.6, 0.7, 0.8, 1). When constructing the decision tree, these parameters are random within the defined range. The number of folds for cross-validation is 5. For the support vector machine algorithm, we chose the radial basis function as the kernel function. Different from the two algorithms, linear regression does not require any parameters while performing machine learning.

## Supplementary Information

Supplementary Figures.

## Data Availability

The machine learning data and codes that support the findings of this study are available from the corresponding author upon request.
