# Supplementary material for: Efficient machine learning of solute segregation energy based on physics-informed features
Source: Sci Rep. 2023 Jul 15;13:11449. doi: 10.1038/s41598-023-38533-8 (PMC10349884; doi:10.1038/s41598-023-38533-8)
Supplement: Supplementary file 1 — Supplementary Figures. [file 41598_2023_38533_MOESM1_ESM.docx]

**Supplementary Information for**

**Efficient Machine Learning of**

**Solute Segregation Energy Based on Physics-informed Features**

Zongyi Ma and Zhiliang Pan^*^

Guangxi Education Department Key Laboratory of Microelectronic Packaging & Assembly Technology, School of Mechanical & Electrical Engineering, Guilin University of Electronic Technology, Guilin 541004, China

*To whom correspondence should be addressed: [zpan@guet.edu.cn](mailto:zpan@guet.edu.cn)

**Table of Content:**

1. Supplementary Figures S1-S6


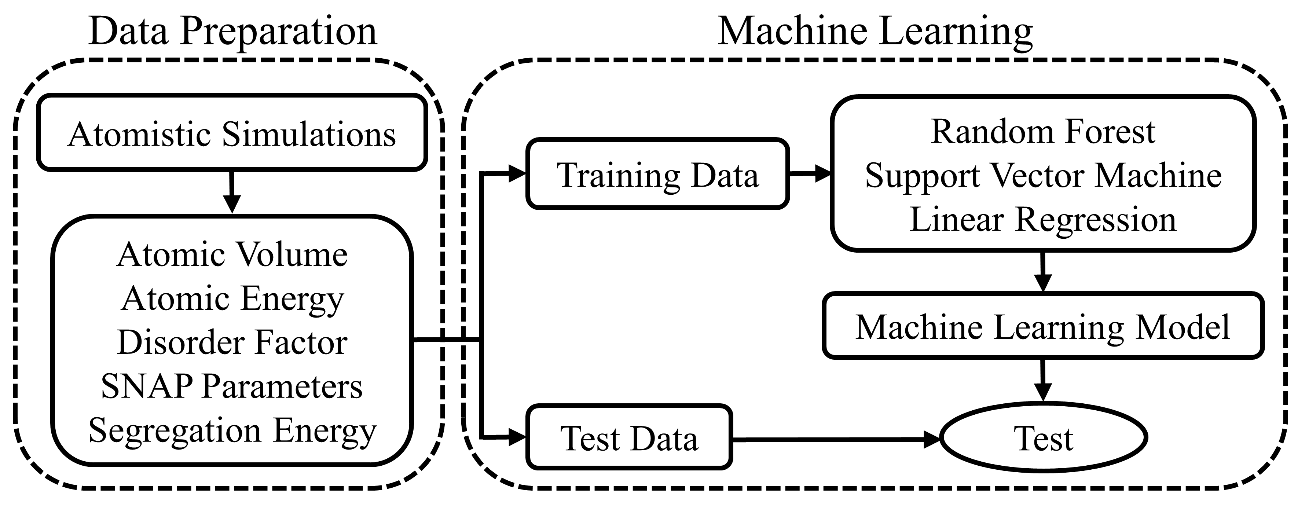


**Fig. S1. Machine learning modeling process of solute segregation energy.** Three general steps, data preparation using atomistic simulations, model training using three algorithms, and corresponding model testing are included in the whole process.


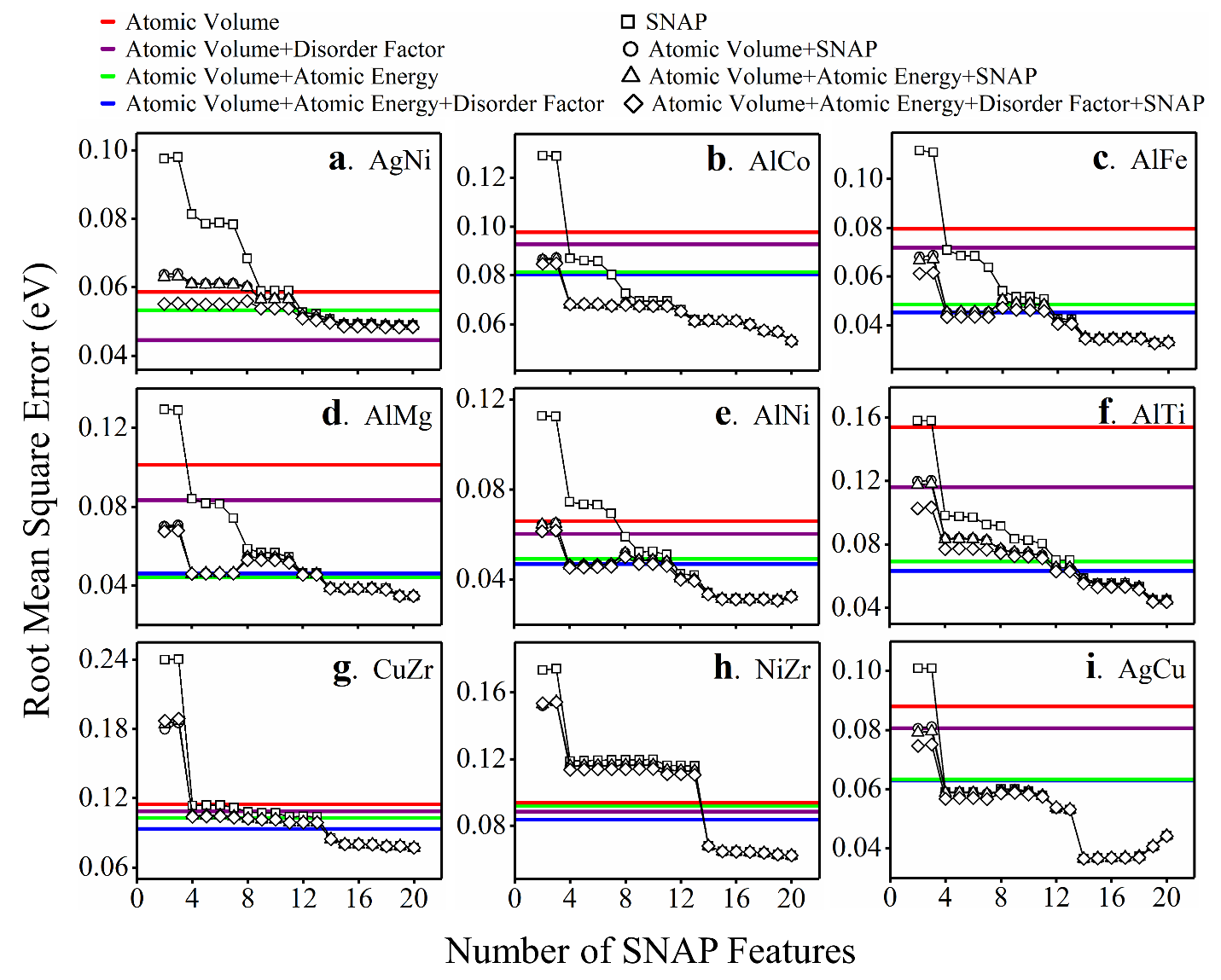


**Fig. S2. The performance of PI features on nine alloy systems (a-i) using support vector machine algorithm**. PI features are much more accurate than the same number of SNAP features while used separately, significantly decrease the number of SNAP features for the same accuracy while combined with SNAP features, and have no overfitting issues that the SNAP features suffer a lot.


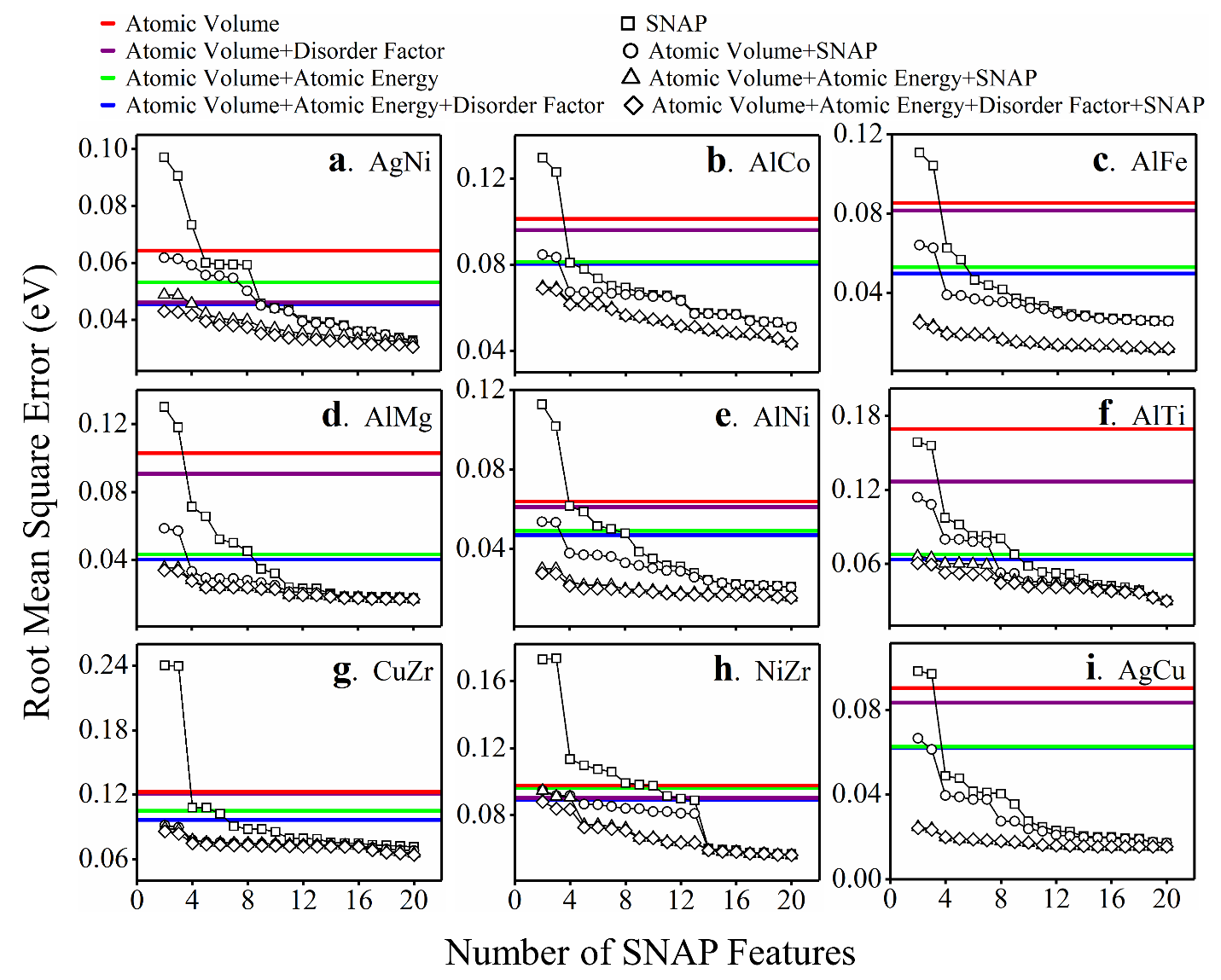


**Fig. S3. The performance of PI features on nine alloy systems (a-i) using linear regression algorithm**. PI features are much more accurate than the same number of SNAP features while used separately and significantly decrease the number of SNAP features for the same accuracy when combined with SNAP features.


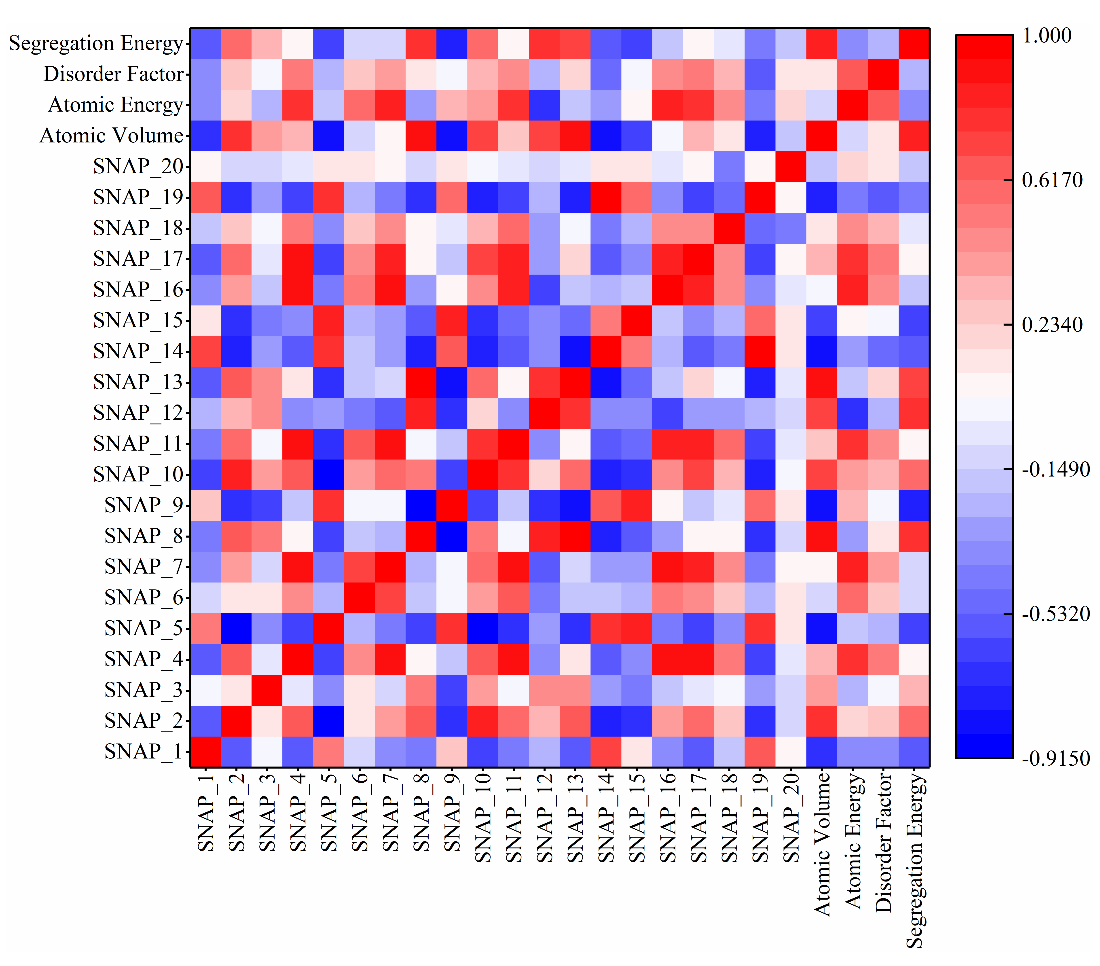


**Fig. S4. Correlation matrix heat map of SNAP & PI features and the Ni segregation energy in Ag polycrystalline GB network.** The correlation between the three PI features is relatively weak, except that the correlation between atomic energy and disorder factor is stronger, indicating that disordered structure tends to have high energy.


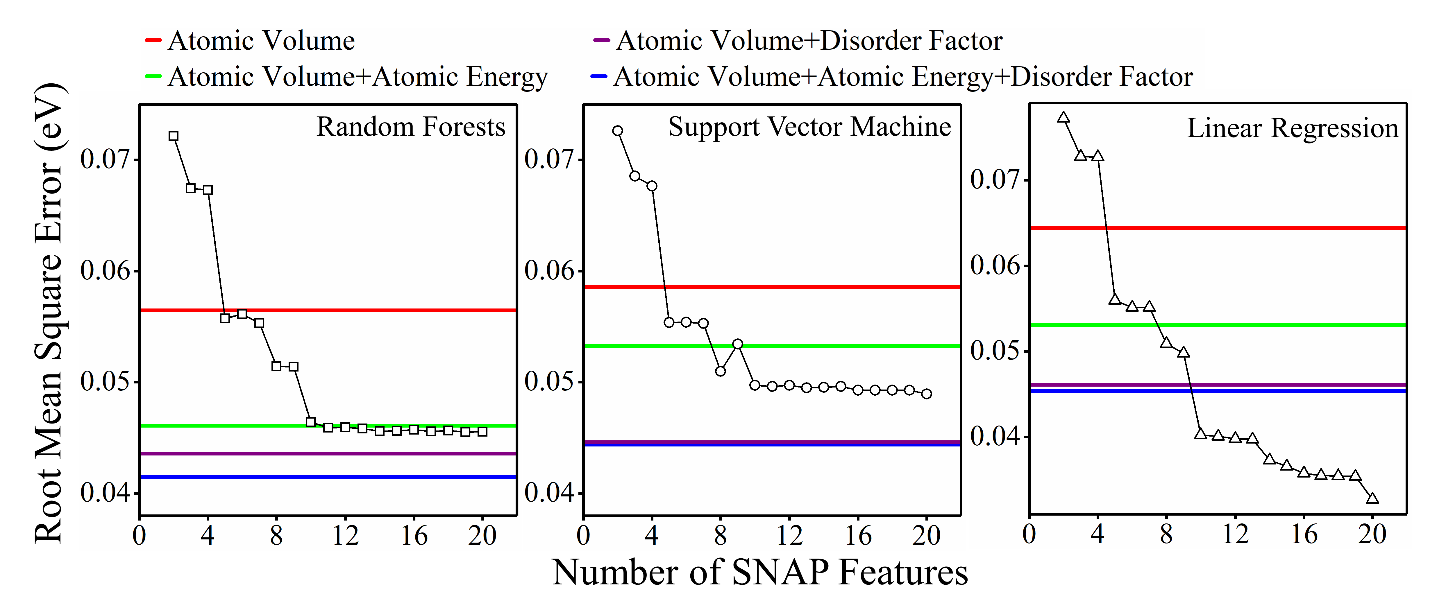


**Fig. S5. The performance of PI features on the AgNi system with SNAP features sorted from the greatest to least relevance to segregation energy.** PI features are still more accurate than the same number of SNAP features while used separately and significantly decrease the number of SNAP features for the same accuracy while combined with SNAP features.


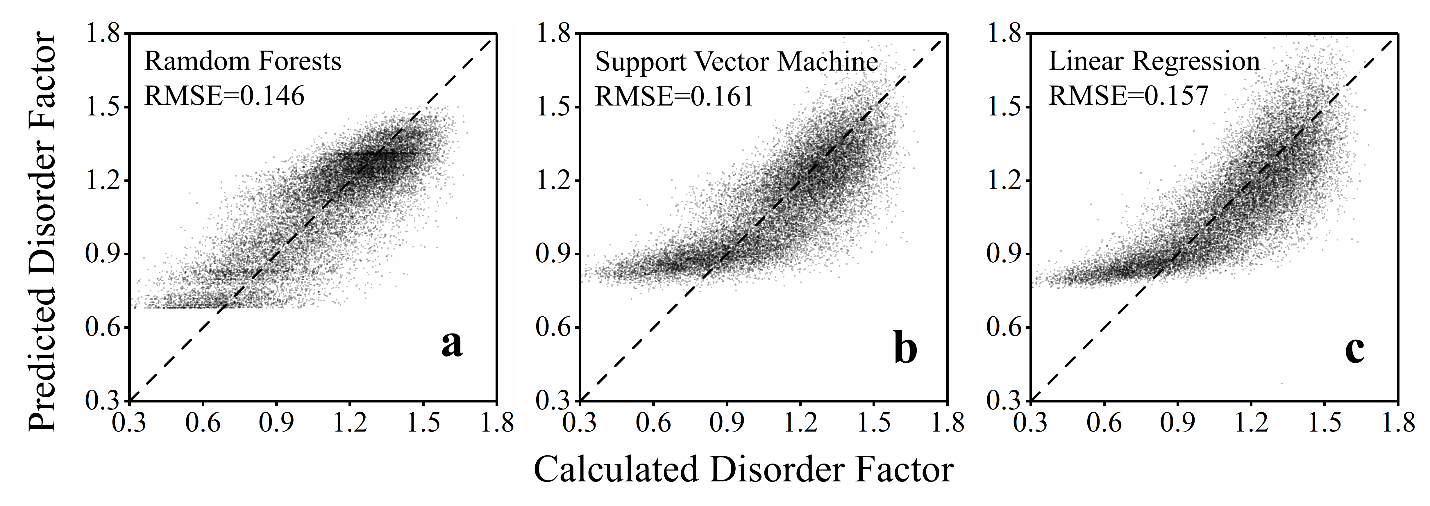


**Fig. S6. Validation of machine learning modeling of disorder factor based on SNAP features in AgNi alloy.** The three machine learning algorithms (**a-c**) all give a relatively high RMSE, indicating that the information on the disorder factor is not sufficiently contained in the SNAP features. This is a typical example showing that the SNAP features lose information while extracted from the local atomic environment.
